# Variations in different polycystic ovary syndrome phenotypes in a population of Chinese Han

**DOI:** 10.1530/EC-13-0080

**Published:** 2014-03-01

**Authors:** Rong Huang, Jun Zheng, Shengxian Li, Lihua Wang, Tao Tao, Xiangyu Teng, Jing Ma, Wei Liu

**Affiliations:** 1 Department of Endocrinology and Metabolism, School of Medicine, Ren Ji Hospital, Shanghai Jiao Tong University, 160 Pujian Road, Shanghai 200127, People's Republic of China

The above article has been retracted. The retraction was made before the article reached its final form in the publication process. However, the authors’ accepted manuscript, prior to copy editing, page layout and proofing, was made available online as an *Endocrine Connections* Accepted Preprint on 20 November 2013.

The article was submitted to the journal in error and retracted at the request of the authors.

